# Supplementary material for: Rank Normalization Enables Cross Pathway Comparison in Degradation Resolved Proteome Turnover Analysis
Source: bioRxiv. 2026 Oct 1:2026.09.30.755414. Preprint. [Version 1] doi: 10.64898/2026.09.30.755414 (PMC13648728; doi:10.64898/2026.09.30.755414)
Supplement: Supplementary file 1 [file NIHPP2026.09.30.755414V1-supplement-1.pdf]

bioRxiv preprint doi: https://doi.org/10.64898/2026.09.30.755414; this version posted October 2, 2026. The copyright holder for this preprint (which was not certified by peer review) is the author/funder, who has granted bioRxiv a license to display the preprint in perpetuity. It is made available under a CC-BY 4.0 International license.

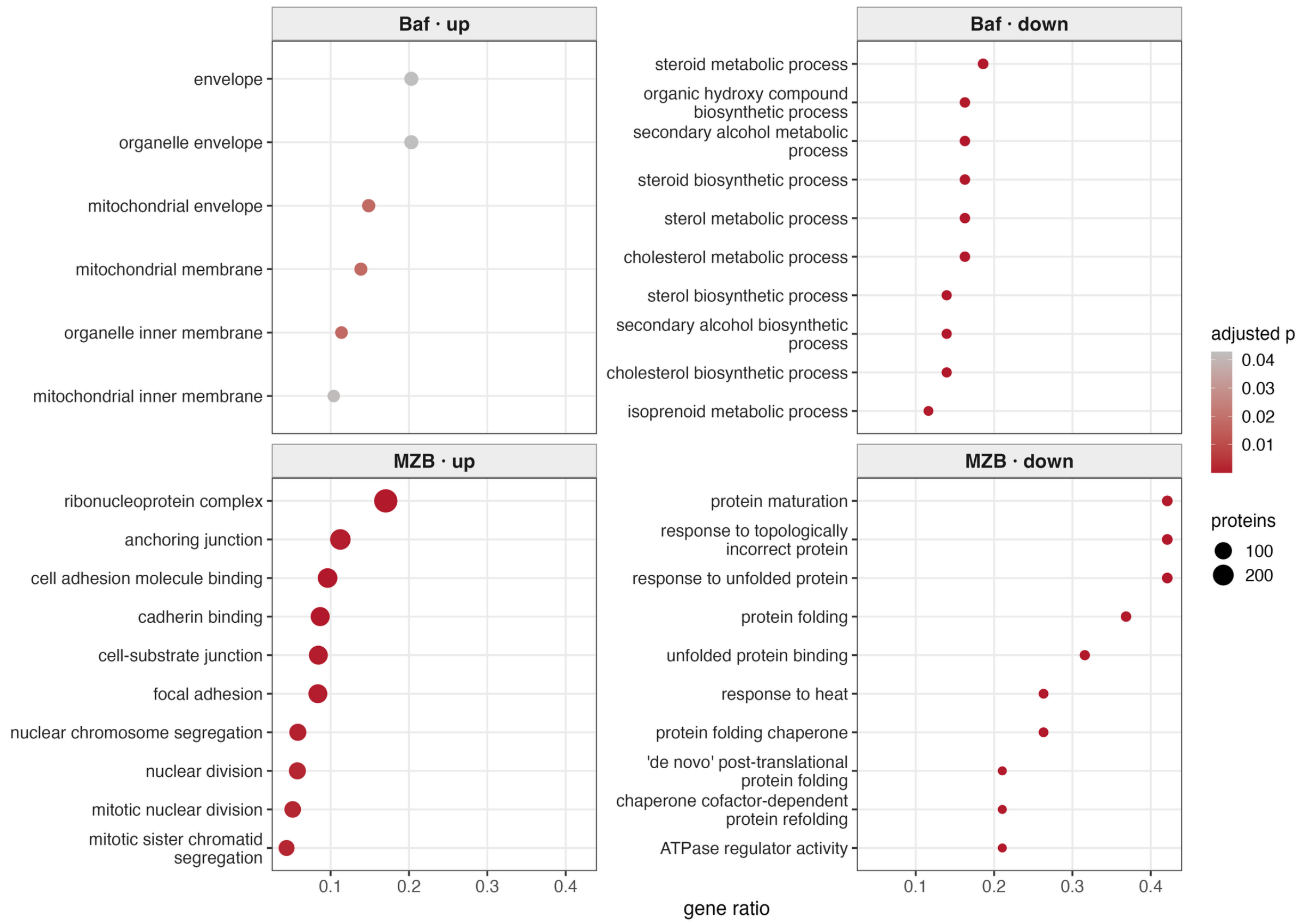

Figure S1. Gene ontology overrepresentation among proteins with altered apparent half-life under each perturbation. Terms enriched among proteins stabilized (up) or destabilized (down) by BafA1 and MZB were tested separately against the quantified proteome. Dot size represents the number of annotated proteins, color indicates the Benjamini-Hochberg adjusted p value, and the x axis shows gene ratio.

bioRxiv preprint doi: https://doi.org/10.64898/2026.09.30.755414; this version posted October 2, 2026. The copyright holder for this preprint (which was not certified by peer review) is the author/funder, who has granted bioRxiv a license to display the preprint in perpetuity. It is made available under a CC-BY 4.0 International license.

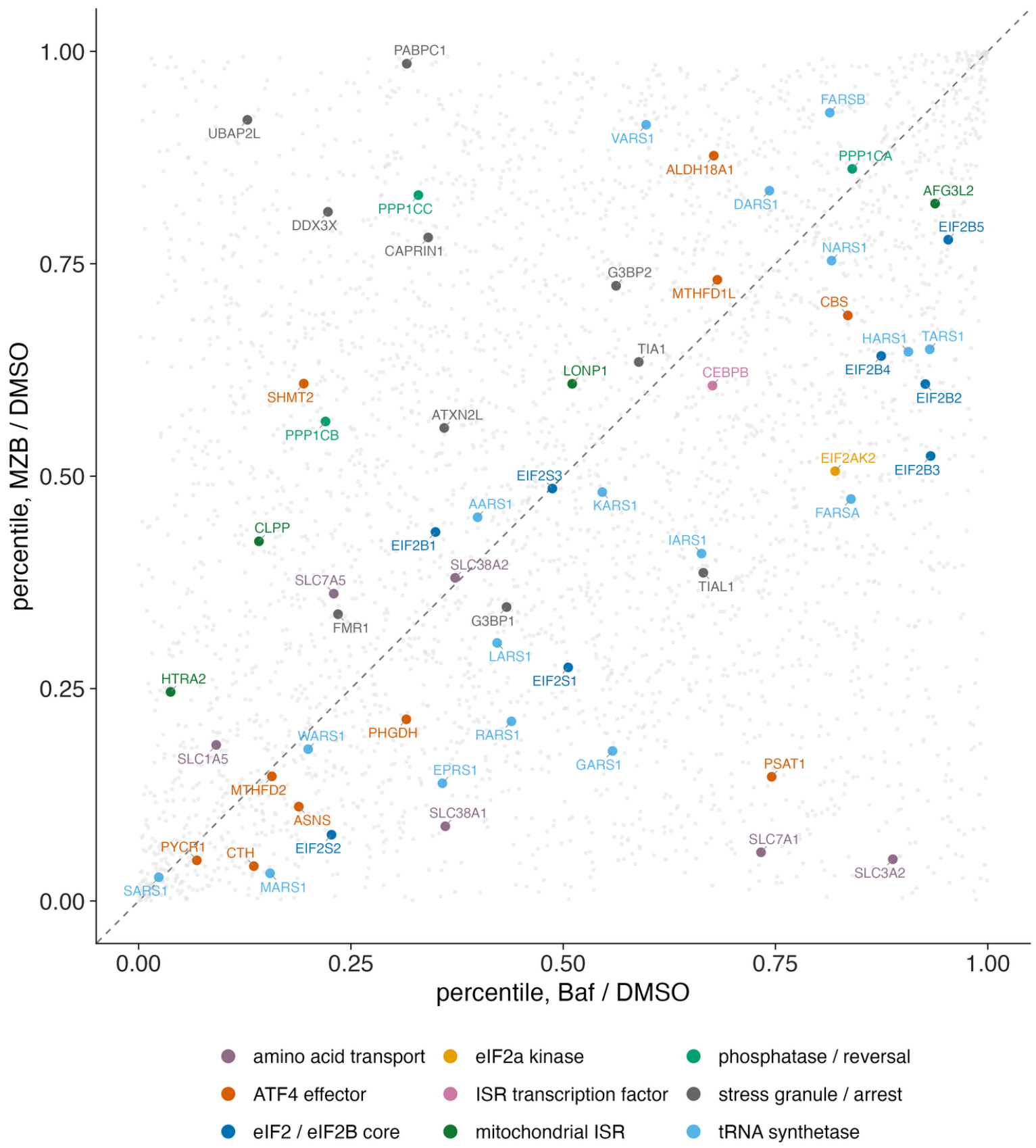

Figure S2. Integrated stress response proteins do not occupy a single region of the degradation preference map. Percentile responses under MZB and BafA1 are shown for manually curated ISR associated proteins overlaid on the full quantified proteome in grey. Points are grouped by functional category and labeled with gene symbols. The dashed line is the identity diagonal.

bioRxiv preprint doi: https://doi.org/10.64898/2026.09.30.755414; this version posted October 2, 2026. The copyright holder for this preprint (which was not certified by peer review) is the author/funder, who has granted bioRxiv a license to display the preprint in perpetuity. It is made available under a CC-BY 4.0 International license.

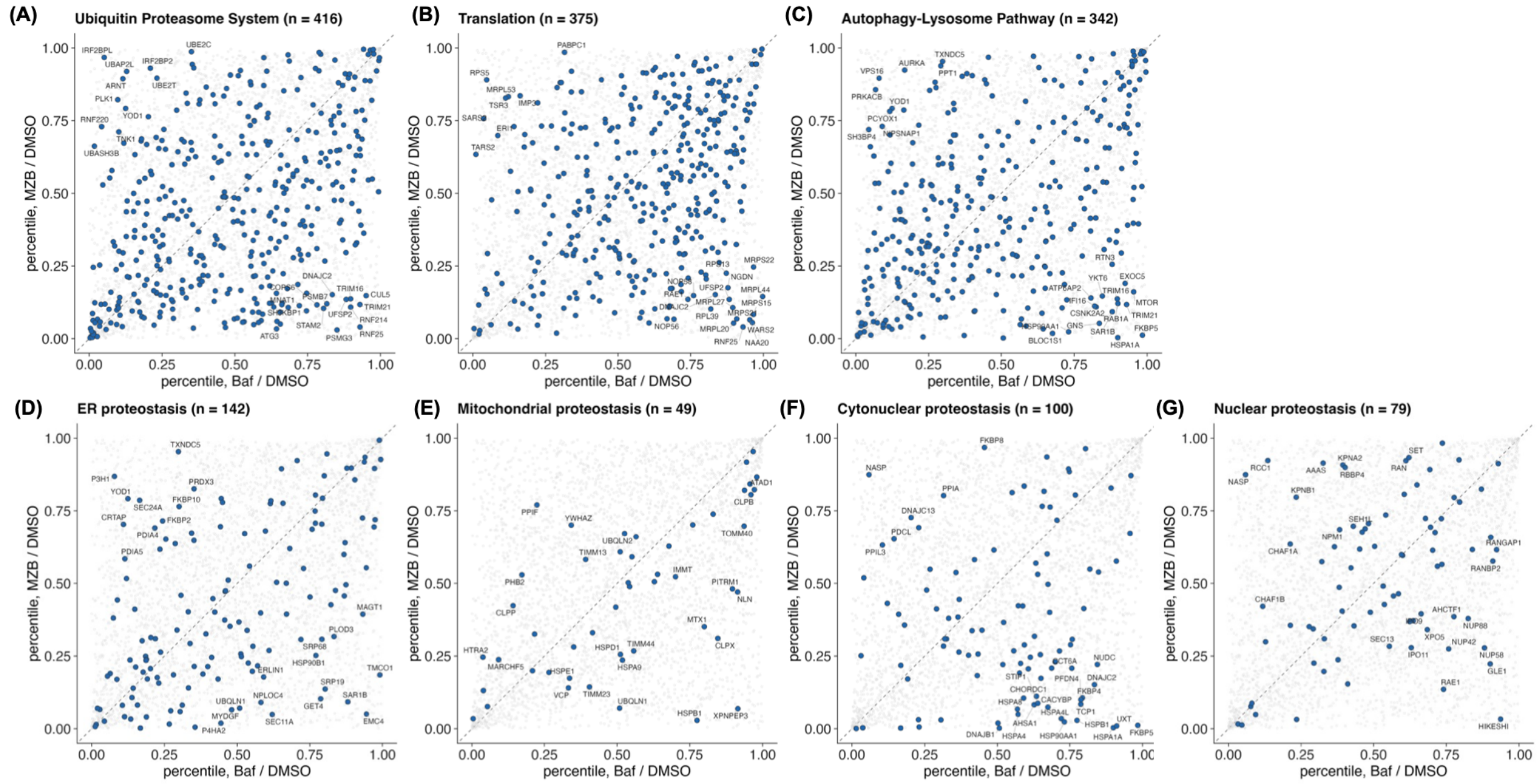

Figure S3. Degradation preference maps for individual proteostasis network branch. Percentile responses under MZB and BafA1 are shown for members of each PN class (A to G) overlaid on the full quantified proteome in grey. The class name and number of quantified members are given in each panel title. Point color indicates the direction of the class level sign test, with blue indicating ALP preference and red indicating UPS preference. The dashed line is the identity diagonal, and individual proteins are labeled with gene symbols.

bioRxiv preprint doi: https://doi.org/10.64898/2026.09.30.755414; this version posted October 2, 2026. The copyright holder for this preprint (which was not certified by peer review) is the author/funder, who has granted bioRxiv a license to display the preprint in perpetuity. It is made available under a CC-BY 4.0 International license.

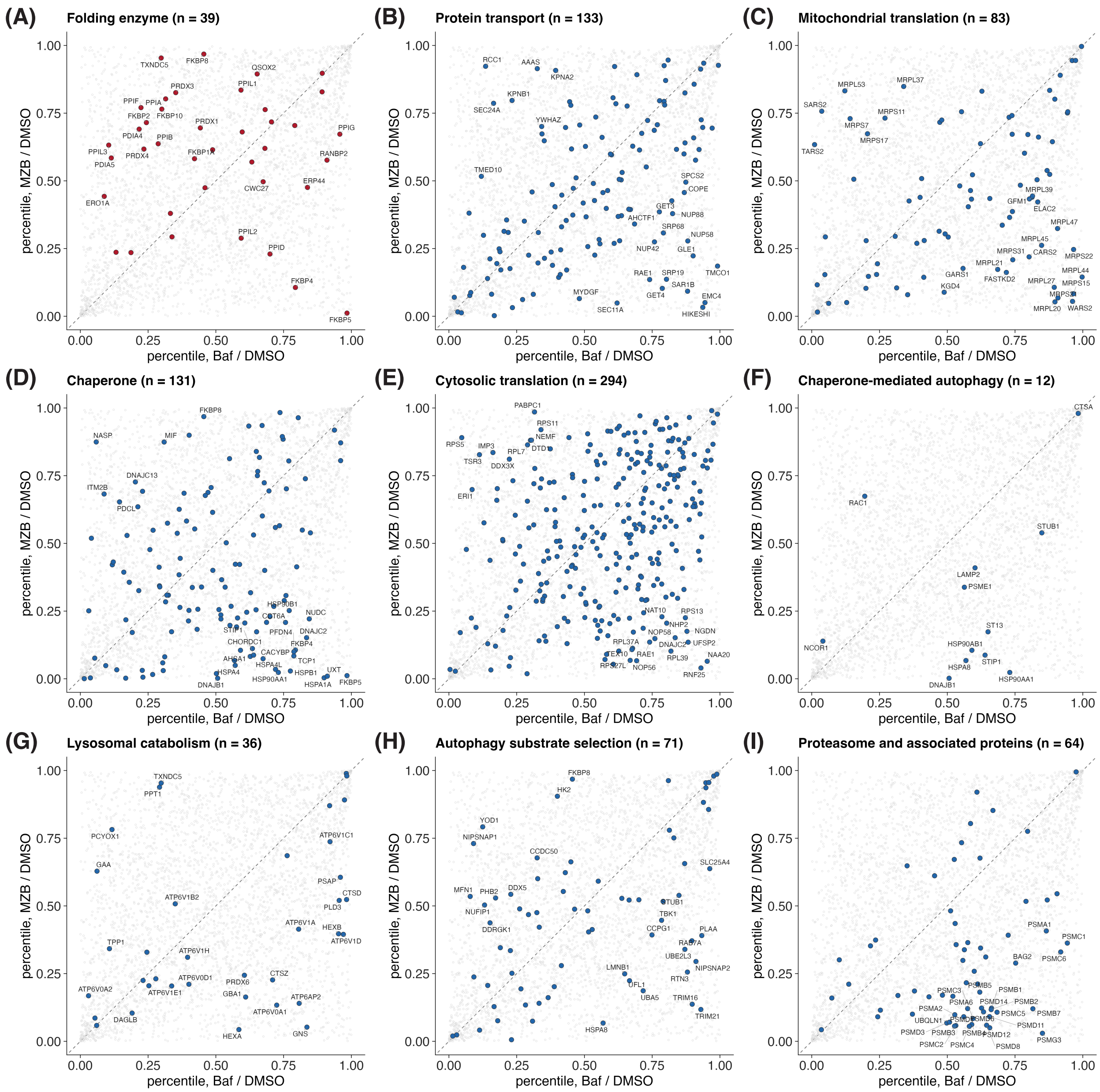

Figure S4. Degradation preference maps for individual proteostasis network classes. Percentile responses under MZB and BafA1 are shown for members of each PN class (A to I) overlaid on the full quantified proteome in grey. The class name and number of quantified members are given in each panel title. Point color indicates the direction of the class level sign test, with blue indicating ALP preference and red indicating UPS preference. The dashed line is the identity diagonal, and individual proteins are labeled with gene symbols.

bioRxiv preprint doi: https://doi.org/10.64898/2026.09.30.755414; this version posted October 2, 2026. The copyright holder for this preprint (which was not certified by peer review) is the author/funder, who has granted bioRxiv a license to display the preprint in perpetuity. It is made available under a CC-BY 4.0 International license.

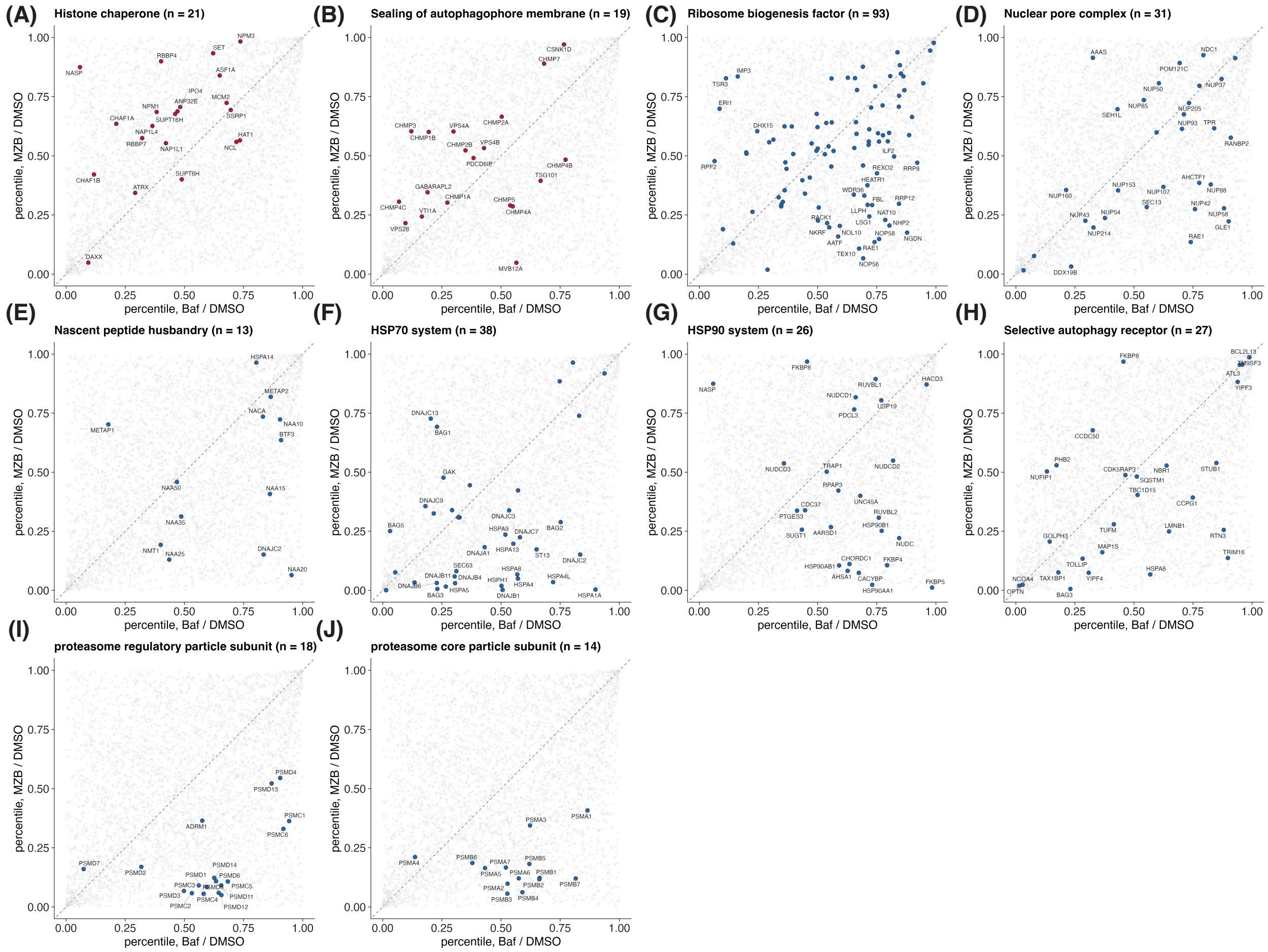

Figure S5. Degradation preference maps for individual proteostasis network groups. Percentile responses under MZB and BafA1 are shown for members of each PN group (A to J) overlaid on the full quantified proteome in grey. The group name and number of quantified members are given in each panel title. Point color indicates the direction of the group level sign test, with blue indicating ALP preference and red indicating UPS preference. The dashed line is the identity diagonal, and individual proteins are labeled with gene symbols.
